# Protein-Tyrosine Kinase Activity Profiling in Knock Down Zebrafish Embryos

**DOI:** 10.1371/journal.pone.0000581

**Published:** 2007-07-04

**Authors:** Simone Lemeer, Chris Jopling, Faris Naji, Rob Ruijtenbeek, Monique Slijper, Albert J.R. Heck, Jeroen den Hertog

**Affiliations:** 1 Hubrecht Institute, Utrecht, The Netherlands; 2 Department of Biomolecular Mass Spectrometry, Bijvoet Center for Biomolecular Research and Utrecht Institute for Pharmaceutical Sciences, Utrecht University, Utrecht, The Netherlands; 3 Pamgene International B.V., Hertogenbosch, The Netherlands; University of Washington, United States of America

## Abstract

**Background:**

Protein-tyrosine kinases (PTKs) regulate virtually all biological processes. PTKs phosphorylate substrates in a sequence-specific manner and relatively short peptide sequences determine selectivity. Here, we developed new technology to determine PTK activity profiles using peptide arrays. The zebrafish is an excellent model system to investigate signaling in the whole organism, given its wealth of genetic tools, including morpholino-mediated knock down technology. We used zebrafish embryo lysates to determine PTK activity profiles, thus providing the unique opportunity to directly compare the effect of protein knock downs on PTK activity profiles on the one hand and phenotypic changes on the other.

**Methodology:**

We used multiplex arrays of 144 distinct peptides, spotted on a porous substrate, allowing the sample to be pumped up and down, optimizing reaction kinetics. Kinase reactions were performed using complex zebrafish embryo lysates or purified kinases. Peptide phosphorylation was detected by fluorescent anti-phosphotyrosine antibody binding and the porous chips allowed semi-continuous recording of the signal. We used morpholinos to knock down protein expression in the zebrafish embryos and subsequently, we determined the effects on the PTK activity profiles.

**Results and Conclusion:**

Reproducible PTK activity profiles were derived from one-day-old zebrafiish embryos. Morpholino-mediated knock downs of the Src family kinases, Fyn and Yes, induced characteristic phenotypes and distinct changes in the PTK activity profiles. Interestingly, the peptide substrates that were less phosphorylated upon Fyn and Yes knock down were preferential substrates of purified Fyn and Yes. Previously, we demonstrated that Wnt11 knock down phenocopied Fyn/Yes knock down. Interestingly, Wnt11 knock down induced similar changes in the PTK activity profile as Fyn/Yes knock down. The control Nacre/Mitfa knock down did not affect the PTK activity profile significantly. Our results indicate that the novel peptide chip technology can be used to unravel kinase signaling pathways *in vivo*.

## Introduction

PTKs are essential regulators of virtually all biological processes and they have pivotal roles in development and disease [1]. PTKs have sequence-specific kinase activities and relatively short peptides have been used to establish the selectivities of large panels of kinases [2]. This feature of kinases, combined with the recent development of peptide array chip technology has allowed researchers to rapidly produce substrate profiles for individual kinases. Moreover, these peptide arrays have been used to determine PTK activity profiles in whole cell lysates [3]–[6]. The PTK activity profiles provide insight into which PTKs are active in the corresponding cells and therefore these PTK activity profiles may be used to dissect PTK signaling pathways. For instance, lipopolysaccharide activation of peripheral blood mononuclear cells induced the phosphorylation of a subset of peptide substrates on the microarray chip, suggesting activation of several kinases, which was confirmed by immunoblotting [4].

To date, kinase activity profiling was done using peptide arrays spotted onto glass coverslips. These are incubated with a kinase (mixture) and radioactively labeled ATP, [γ-^33^P]ATP. Detection of phosphorylation is done by autoradiography, using phosphoimaging technology for quantification [4]–[6]. We have developed novel non-radioactive technology using peptides encoding known phosphorylation sites as substrates spotted onto porous chips [7], [8]. The use of porous chips allowed the kinase reaction samples to be pumped up and down, optimizing reaction kinetics. Peptide phosphorylation was detected by fluorescent anti-phosphotyrosine antibody binding and the porous chips allowed semi-continuous recording of the signal.

We have used the PTK activity profiling described above for purified PTKs. Moreover, we determined PTK activity profiles in complex mixtures of zebrafish embryo lysates. The zebrafish is increasingly being used as a model for human disease [9], [10]. Moreover, zebrafish embryos are an excellent model system for functional analysis of molecular signaling in whole organisms, because the embryos are easily accessible for experimentation and for morphological analysis of phenotypes. Specific, targeted knock down of protein expression using morpholinos in zebrafish embryos allows for rapid assessment of phenotypic and molecular changes [11], [12].

Here, we report that peptide chip arrays allowed us to reproducibly determine PTK activity profiles in 1 day old zebrafish embryos. Knock down of the Src family kinases (SFKs), Fyn and Yes, led to specific changes in the PTK activity profile. Moreover, knock down of the non-canonical Wnt11 that induced similar phenotypes as the Fyn and Yes knock downs [13] resulted in similar changes in the PTK activity profile. Our results demonstrate that PTK activity profiling may be used to unravel PTK signaling in whole organisms.

## Results and Discussion

**Figure 1:**
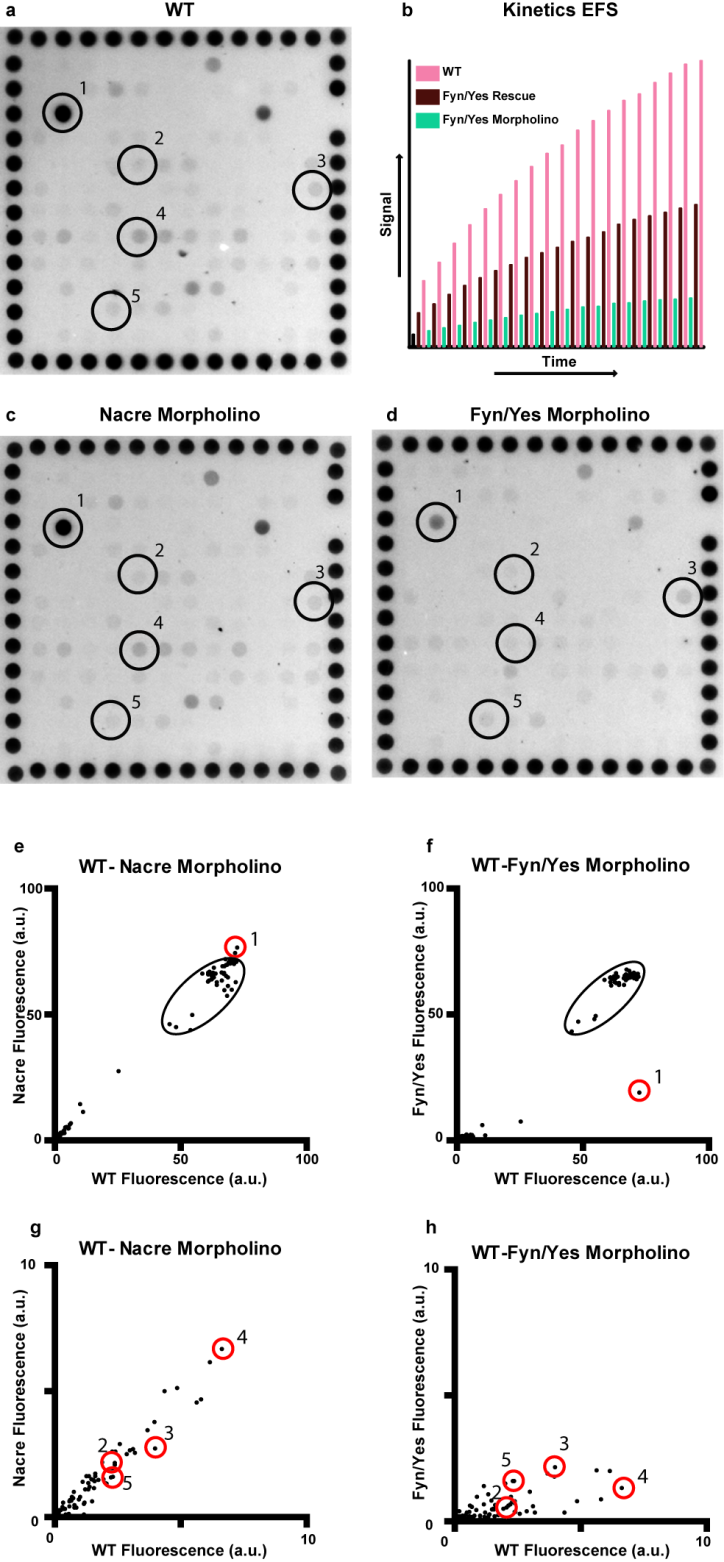
PTK profiles of 24 hpf zebrafish embryos. (a) Fluorescence of the panel of 144 peptides is shown after 60 min (endpoint). The fluorescently labeled control peptides at the periphery indicate the grid of 144 different peptides. (b) Kinetics of EFS peptide phosphorylation by control embryo lysate (pink), Fyn/Yes morpholino injected embryo lysate (green) and Fyn/Yes morpholino, Fyn/Yes RNA co-injected embryos (Fyn/Yes rescue, brown) is shown with a data point every three min and the fluorescence in arbitrary units. (c,d) Endpoint measurements of peptide phosphorylation by Nacre- or Fyn/Yes morpholino-injected embryo lysates. (e–h) Quantification of PTK profiles. The relative intensities of peptide phosphorylation by lysates of control, non-injected embryos were plotted against Nacre- or Fyn/Yes knock down embryos. The fluorescently labeled reference spots all lie within the indicated oval. (g,h) are a close-up of the low range of panels e and f. The five highlighted peptides are: 1,EFS; 2, FAK2; 3, LTK; 4, PAXI and 5, SYN1.

Here, we have used peptide array technology for the first time in zebrafish embryos to determine PTK activity profiles. The peptide arrays consist of 144 peptides, each 15 amino acids in length covering known phosphorylation sites (Table S1). Twenty zebrafish embryos were de-yolked, pooled and lysed at 24 hour post fertilization (hpf). The equivalent of two to four lysed embryos was used to determine PTK activities, demonstrating high sensitivity of the assay. Since we pooled twenty embryos per data point, subtle differences between individual embryos were not detected in our assays. The kinase reaction samples were pumped up and down through the porous chips, optimizing reaction kinetics. The fluorescent signal was recorded semi-continuously every 3 min for 60 min. Characteristic, reproducible PTK profiles were derived using non-injected control zebrafish embryo lysates (Fig. 1a). The fluorescence intensities were quantified for each peptide (Table S1) and the fluorescent peptides at the periphery of the specific peptide grid were included as a detection control. Kinetic analysis for each peptide demonstrated that the signals were not saturating yet, not even for the most highly phosphorylated EFS peptide (Fig. 1b).

**Figure 2:**
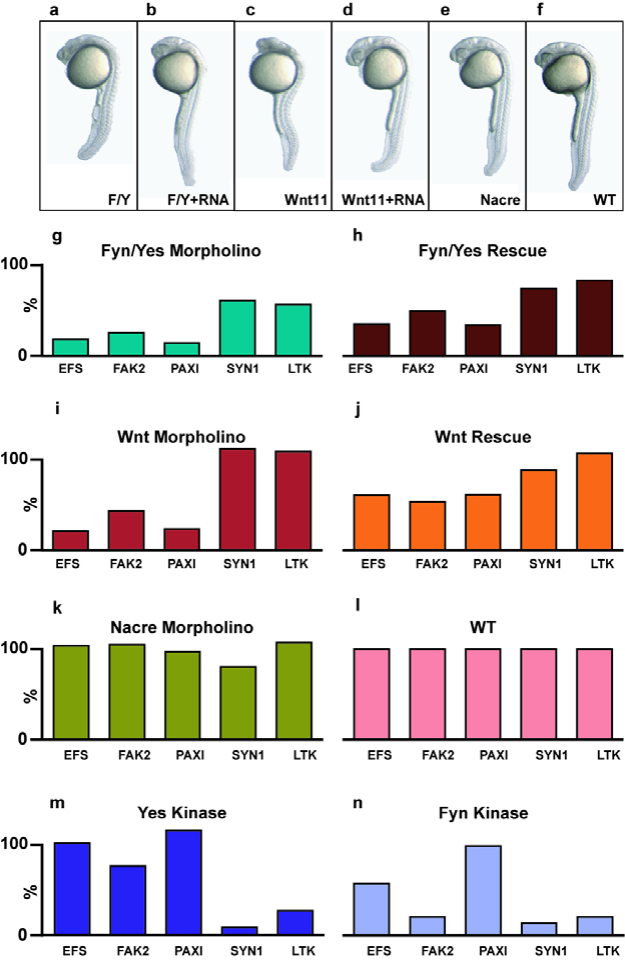
Similarities in PTK activity profiles of Fyn/Yes and Wnt11 knock downs. (a–f) Morphology of 24 hpf zebrafish embryos after knock down of Fyn/Yes (F/Y), Wnt11 or Nacre and of recues of the indicated knock downs using the corresponding synthetic RNAs. (g–l) Relative intensities of phosphorylation of a selection of five peptides. The signal was determined in duplicate and calculated relative to non-injected wild type control (set to 100%). (m–n) Relative intensities of phosphorylation of the same set of five peptides by purified Yes and Fyn kinases.

Next, we determined PTK activity profiles following morpholino-mediated knock down of the Src family kinases (SFKs), Fyn and Yes. Previously, we have shown that Fyn/Yes knock downs affect convergence and extension (CE) cell movements [13]. As a control, we used the Nacre/Mitf morpholino that does not induce phenotypes similar to the Fyn/Yes knock downs, but only affects pigmentation [11]. The PTK profile of 24 hpf Nacre knock down embryos was highly similar to the PTK profile of wild type embryos (Fig. 1c,e,g). However, the PTK profile from Fyn/Yes knock down embryos differed significantly from the PTK profile of wild type embryos. The intensity of several spots was dramatically reduced upon Fyn/Yes knock down (Fig. 1d,f,h). The most prominently phosphorylated peptide in 24 hpf embryos was derived from embryonic Fyn substrate (EFS, spot #1 in Fig. 1) and phosphorylation of this peptide was clearly reduced upon Fyn/Yes knock down. The reference peptides displayed similar fluorescence intensities from chip to chip (Fig. 1e,f). The kinase activities towards all the peptides on the chip in wild type and knock down embryos are depicted in Table S1. Our results indicate that Fyn/Yes knock down induced a significant decrease in kinase activity towards a subset of the peptides on the chip, suggesting that this reduction is specific. For clarity, we selected a small panel of peptides that were less phosphorylated by the Fyn/Yes knock down embryo lysates (derived from EFS, FAK2 and Paxilin, respectively #1, 2 and 4). As a negative control, we selected two peptides that were much less affected by Fyn/Yes knock down (derived from Ltk or Syn1, #3 and 5). PTK profiles of purified Fyn and Yes proteins indicated that indeed FAK2, Paxillin and EFS were preferred substrates of Fyn and Yes, whereas Ltk and Syn1 were poorly phosphorylated by these SFKs (Fig. 2m,n). These results indicate that reduced phosphorylation of peptides in Fyn/Yes knock down embryos is directly attributable to reduced expression of Fyn and Yes in the knock down embryos. However, we cannot exclude the possibility that other, more downstream kinases with overlapping specificities contribute to the observed effects.

To ensure that the Fyn/Yes knock down-induced differences in PTK profile were specific, Fyn/Yes morpholinos and synthetic RNA encoding Fyn and Yes were co-injected at the one-cell stage. Consistent with our previous results, these co-injections effectively rescued the Fyn/Yes knock down phenotype (Fig. 2a,b). Co-injection of Fyn and Yes RNA led to an increase in phosphorylation of most peptides that show reduced phosphorylation upon Fyn/Yes knock down, including FAK2, Paxillin and EFS (Fig. 2g,h, Table S1). Hence, Fyn and Yes RNA induced at least a partial rescue of the PTK profile in 24 hpf embryos. The Syn1 and LTK peptides that were much less affected by Fyn/Yes knock down in the first place, were not affected by co-injection of Fyn and Yes RNA (Fig. 2g,h).

**Figure 3:**
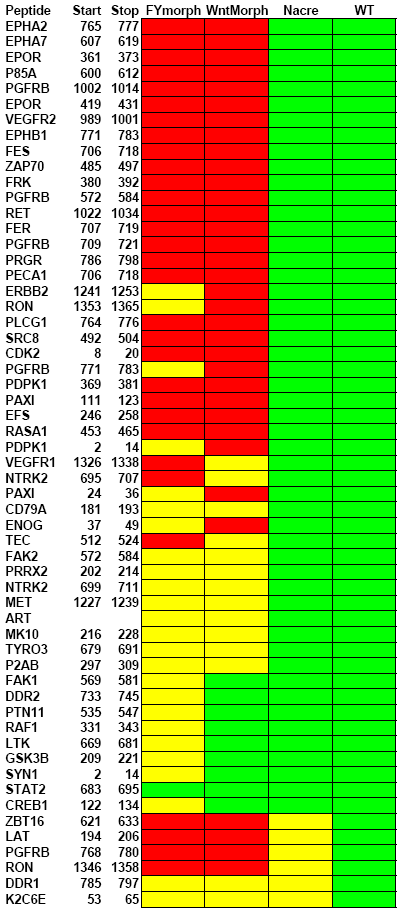
Peptide phosphorylation profiles of Fyn/Yes and Wnt11 knock down embryos are largely overlapping. Peptide phosphorylation of Fyn/Yes (FYmorph), Wnt11 (Wntmorph) or Nacre knock down was determined relative to wild type (WT), which was set to 100%. The average of a duplicate experiment is depicted here. Phosphorylation intensities were color coded, <25%, red; 25–75%, yellow and 75–125%, green. Protein identities of the peptides are indicated as well as their start and end residues. The sequence of the peptides and numerical representation of the data is given in Table S1. The peptides are listed from most affected at the top to least affected at the bottom. Phosphorylation of the bottom six peptides is also reduced upon injection of the control Nacre morpholino and these may represent non-specific effects.

Previously, we have shown that Fyn and Yes operate in a pathway that converges with non-canonical Wnt signalling to regulate convergent extension cell movements during vertebrate gastrulation (Fig. 2 a–d)[13]. Therefore, we wondered whether Wnt11 knock down embryos show a similar PTK profile as Fyn/Yes knock downs. Indeed, many peptides, including FAK2, Paxillin and EFS were significantly less phosphorylated upon Wnt11 knock down. Co-injection of Wnt11 RNA largely rescued these effects, confirming the specificity of these results (Fig. 2f,j). Comparison of relative peptide phosphorylation of all peptides that were phosphorylated significantly indicated that Fyn/Yes and Wnt11 morpholinos induced largely overlapping changes in PTK activity profiles (Fig. 3). However, a subset of the peptides was phosphorylated normally by the Wnt11 knock down embryo lysates, whereas phosphorylation by the Fyn/Yes knockdown embryo lysates was reduced to some extent. We conclude that the PTK activity profiles after Fyn/Yes and Wnt11 knock down were similar, but not identical. Based on previous results [13], we postulated that Fyn/Yes and non-canonical Wnt signaling converged on RhoA, downstream in the signaling pathway [14], which was consistent with data from *C. elegans* [15]. Here we show that the PTK activity profiles are similar upon knock down of Fyn/Yes and Wnt11, indicating that Fyn/Yes and Wnt11 signaling are required for the activation of largely overlapping panels of PTKs. It is possible that Wnt11 signaling is required for full activation of Fyn and Yes PTK activities. Alternatively, the PTK activity profiles are partially due to kinases that are activated in response to both Fyn/Yes signaling as well as Wnt11 signaling, downstream of RhoA. Future research will be aimed at distinguishing between these two possibilities.

Here we show that it is feasible to produce effective PTK activity profiles of zebrafish embryos by utilising peptide chip technology. Moreover, we demonstrate that the system is amenable to functional assays. We detected significant, reproducible changes in PTK activity profiles from morpholino knock down embryos that were partially rescued by co-injection of the corresponding mRNAs. This technology allows for the rapid identification of candidate proteins/signaling pathways associated with specific genes.

## Material and Methods

### Zebrafish embryos and micro-injections

Zebrafish were kept and the embryos were staged as described before [16]. Antisense morpholinos were ordered from GeneTools (Philomath, OR, USA). The morpholinos were targeted close to the start ATG of the respective cDNAs and their sequences have been described before [13]. The Fyn and Yes morpholinos (4 ng each), Wnt11 morpholino (8 ng) or Nacre morpholino (4 ng) were injected at the one-cell stage. For the rescues, co-injections were done with synthetic RNA, encoding the corresponding gene. Titrations were done with a range of synthetic RNA to obtain optimal rescue of the phenotype.

### De-yolking and lysis

Embryos (24 hpf) were deyolked with deyolking buffer (½ Ginzburg Fish Ringer) without calcium [17]. Subsequently, embryos were lysed in buffer containing 50 mM Tris pH 7.5, 150 mM NaCl, 1 mM sodium orthovanadate, 1% NP-40, 0.1% sodium deoxycholate and EDTA-free protease inhibitor cocktail (Sigma) and homogenized. Lysates were centrifuged at 14,000 g to pellet cellular debris.

### PTK assays

Micro-array experiments were performed using PamChip peptide arrays run on a PamStation4 instrument (PamGene, 's Hertogenbosch, the Netherlands). Four temperature controled peptide chips were run in parallel by pumping the sample up and down through the 3-dimensional porous chip. Data was captured by real-time imaging of the fluorescence signal by CCD imaging [7], [8]. The tyrosine kinase PamChip arrays comprised 144 different peptides (PamGene, 's Hertogenbosch, the Netherlands). Each peptide represents a 15 amino acid sequence, of which 13 residues are derived from a known phosphorylation site from Swissprot and Phosphobase databases (Table S1). Two N-terminal residues link the phosphosite sequence to the solid support of the 3-dimensional chip. The zebrafish embryo lysates were analysed by applying an aliquot of the lysate in kinase reaction buffer (Abl reaction buffer [New England Biolabs], consisting of 100 mM MgCl_2_, 10 mM EGTA, 20 mM DTT and 0.1% Brij 35 in 500 mM Tris/HCl, pH 7.5) containing 12.5 µg/ml fluoresceine labelled PY20 antibody against phosphotyrosine (Exalpha, USA), and 400 µM ATP (Sigma) to the PamChip peptide array. Prior to application of the sample, the chips were blocked using a solution of 2% BSA (Bovine Serum Albumin, Fraction V, Calbiochem, Germany), and washed 2 times using kinase reaction buffer. During 60 minutes incubation at 30°C, real time images were taken automatically every 3 min). Images were analysed by BioNavigator software (PamGene, 's Hertogenbosch, the Netherlands). The fluorescence intensities were expressed as arbitrary units. Relative intensities of individual peptides on the chip were calculated and compared between different zebrafish embryo lysates. They are expressed as percentage of the intensity in wild type, non-injected controls. Purified Fyn and Yes kinases were from Upstate (Millipore, Billerica, MA, USA).

## Supporting Information

Table S1 The positions of the peptides on the chip are indicated as well as their protein names, the amino acid residue numbers and the sequences of the peptides. Fluorescence intensities for all peptides were determined quantitatively and expressed as arbitrary units. Relative intensities were calculated within individual peptide chips to allow for comparison between different experiments. Averages of two independent experiments were determined and are expressed here as relative signal compared to the signal in wild type, non-injected control embryos (set to 100%). Absolute levels of fluorescence are indicated for wild type, non-injected control (arbitrary units). Signals below the detection threshold level are indicated by a -. The fluorescence intensities of the PTK activity profiles of purified Fyn and Yes are depicted in arbitrary units.
(0.05 MB XLS)
